# Live Cell Imaging Reveals Novel Functions of *Salmonella enterica* SPI2-T3SS Effector Proteins in Remodeling of the Host Cell Endosomal System

**DOI:** 10.1371/journal.pone.0115423

**Published:** 2014-12-18

**Authors:** Roopa Rajashekar, David Liebl, Deepak Chikkaballi, Viktoria Liss, Michael Hensel

**Affiliations:** 1 Mikrobiologisches Institut, Universitätsklinikum Erlangen, Erlangen, Germany; 2 Cell Biology and Biophysics Unit, EMBL Heidelberg, Heidelberg, Germany; 3 Abteilung Mikrobiologie, Universität Osnabrück, Osnabrück, Germany; The Biodesign Institute, Arizona State University, United States of America

## Abstract

Intracellular *Salmonella enterica* induce a massive remodeling of the endosomal system in infected host cells. One dramatic consequence of this interference is the induction of various extensive tubular aggregations of membrane vesicles, and tubules positive for late endosomal/lysosomal markers are referred to as *Salmonella*-induced filaments or SIF. SIF are highly dynamic in nature with extension and collapse velocities of 0.4–0.5 µm x sec^−1^. The induction of SIF depends on the function of the *Salmonella* Pathogenicity Island 2 (SPI2) encoded type III secretion system (T3SS) and a subset of effector proteins. In this study, we applied live cell imaging and electron microscopy to analyze the role of individual effector proteins in SIF morphology and dynamic properties of SIF. SIF in cells infected with *sifB*, *sseJ*, *sseK1*, *sseK2*, *sseI*, *sseL*, *sspH1*, *sspH2*, *slrP*, *steC*, *gogB* or *pipB* mutant strains showed a morphology and dynamics comparable to SIF induced by WT *Salmonella.* SIF were absent in cells infected with the *sifA*-deficient strain and live cell analyses allowed tracking of the loss of the SCV membrane of intracellular *sifA Salmonella*. In contrast to analyses in fixed cells, in living host cells SIF induced by *sseF*- or *sseG*-deficient strains were not discontinuous, but rather continuous and thinner in diameter. A very dramatic phenotype was observed for the *pipB2*-deficient strain that induced very bulky, non-dynamic aggregations of membrane vesicles. Our study underlines the requirement of the study of *Salmonella*-host interaction in living systems and reveals new phenotypes due to the intracellular activities of *Salmonella*.

## Introduction

*Salmonella enterica* is a facultative intracellular pathogen that modifies eukaryotic host cells in order to establish a unique parasitophorous vacuole, the *Salmonella*-containing vacuole or SCV. The SCV is a membrane-bound compartment that has several features of late endosomal compartments and allows the survival and replication of *S. enterica* in a variety of mammalian host cell types [1]. Of central importance for a successful intracellular lifestyle of *Salmonella* is the function of the type III secretion system (T3SS) encoded by *Salmonella* Pathogenicity Island 2 (SPI2) [2]. Intracellular *Salmonella* deploy the SPI2-T3SS to translocate a large number of effector proteins across the SCV membrane. Collectively, these effector proteins enable the intracellular proliferation of *Salmonella* and systemic pathogenesis, but also manipulate a wide range of host cells functions [3]. The molecular functions and the interacting proteins of the host cell are unknown for the majority of the SPI2-T3SS effectors, and not all effectors appear to be relevant for the intracellular phenotypes and systemic pathogenesis under conditions so far tested in the literature.

A specific characteristic of *Salmonella*-infected cells is the extensive reorganization of the endosomal system (reviewed in [4]). The formation of extensive tubular aggregations of endosomal membrane vesicle has been observed and these structures have been termed *Salmonella*-induced filaments or SIF [5]. SIF are characterized by the presence of late endosomal/lysosomal membrane proteins such as LAMP1. The most severe intracellular phenotype is mediated by SPI2-T3SS effector SifA. Mutant strains lacking SifA are highly attenuated in systemic virulence and intracellular replication [6]. Bacteria deficient in *sifA* fail to induce SIF and the bacteria loss the SCV membrane during intracellular replication thereby escaping into the cytoplasm [7]. SseF and SseG contribute to the intracellular lifestyle, although the defects in intracellular replication of the corresponding mutant strains are less pronounced compared to *sifA* or SPI2-T3SS deficient strains [8], [9]. SopD2 is an effector further contributing to formation and maintenance of SCV [10], [11]. A role for PipB2 in control of the centrifugal extension of SIF was observed [12] and PipB2 was identified as a linker for kinesin [13]. SseJ has an enzymatic activity and acts as a deacetylase after translocation into host cells [14]. For SseL, a function as deubiquitinase was observed [15], while SteC affects the host cell actin cytoskeleton [16]. In contrast, the contribution of the further effectors to the intracellular lifestyle of *Salmonella* is less well characterized [3].

The initial studies on SIF biogenesis were performed by immuno-staining of cells fixed at various time-points after infection and these analyses led to the model that SIF emerge from continuous aggregations of endosomes into large tubular compartments [7], [17]. This model probably has to be revised by recent analyses of the intracellular fate of *Salmonella* by live cell imaging. We and others found that the formation of SIF is a highly dynamic process in various types of host cells [18], [19]. SIF show rapid extension, branching and contraction in the early phase of intracellular life of *Salmonella*, while at later stages, an extensive network of SIF is formed that is highly reduced in dynamics. The induction of SIF in living host cells and their dynamic properties were entirely dependent on the function of the SPI2-T3SS and the properties of SIF could be clearly distinguished from other tubular organelles that are found in phagocytic cells.

Based on our initial studies, we set out to analyze the role of various effector proteins of the SPI2-T3SS in the induction of SIF in living host cells, dynamics of SIF and their morphology. Our live cell studies, as well as ultrastructural analyses, demonstrate the specific contribution of SifA, SseF, SseG and PipB2 to formation of SIF, while no contribution was observed for the remaining SPI2-T3SS effector proteins.

## Results

### Interpretation of SCV integrity requires live cell analyses

**Figure 1:**
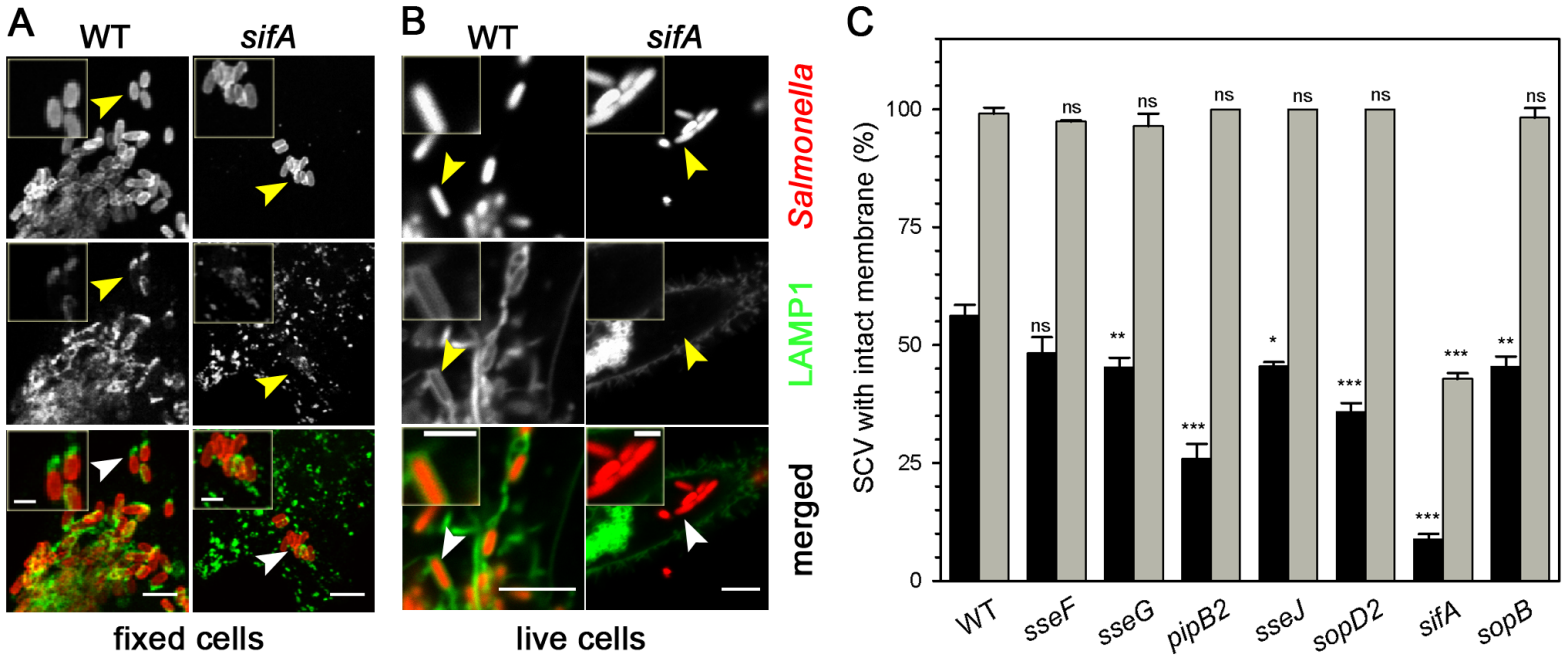
Analysis of the integrity of the *Salmonella*-containing vacuole in living or fixed host cells. For analyses of fixed cells, HeLa cells were infected with *Salmonella* wild-type or various mutant strains. Following incubation for 16 h under standard cell culture conditions, infected cells were fixed with 3% PFA in PBS for 15 min. Immuno-staining was performed for LAMP1 (green) and *Salmonella* O-antigen (red). For analyses of living cells, HeLa cells were transfected with a plasmid for the expression of LAMP1-GFP (green) and infected with *Salmonella* strains expressing mCherry (red). Representative fixed (A) or living cells (B) infected with WT or *sifA* strains were imaged using a Zeiss LSM 700 confocal microscope. Micrographs of live cells infected with further strains are shown in S1 Figure. Arrowheads indicate the positions of individual bacteria and SCV membranes. Scale bars, 5 µm and 1 µm in overview and detail micrographs, respectively. Z sections of live cell infected with WT or *sifA* strains are shown in S2 Figure and animated 3D projections for live cells infected with WT or *sifA* strains are shown in S1 Movie and S2 Movie, respectively. C) For quantitative analyses, at least 20 infected cells per indicated strain were randomly selected for image acquisition and micrographs were used for the analysis of presence or absence of a continuous LAMP1-positive membrane around the bacteria. Black and gray bars represent the number of continuous SCV in fixed and live cells, respectively. Means and standard deviations shown are representative for three independent experiments. Statistical significance between WT and various mutant strains was determined by one-way ANOVA and is indicated as: ns, not significant; *, *P* <0.05; **, *P* <0.01; ***, *P*<0.001.

Our previous live cell-based study showed that SCV, as well as SIF, are complex and dynamic membrane structures [18]. So far, most studies on the role of effector proteins of the SPI2-T3SS in biogenesis and maintenance of the SCV used analyses of fixed cells. In order to investigate the contribution of various effectors to SCV biogenesis and SIF structure and dynamics, we compared the results of conventional analyses using fixed cells to live cell analyses (Fig. 1).

The integrity of the SCV is usually judged based on the presence of late endosomal/lysosomal membrane proteins that are highly abundant in the SCV membrane, as well as in membranes of SIF. LAMP1 is a representative member of the family of lysosomal glycoproteins (lgp) present in late endosomal and lysosomal membranes (reviewed in [20]). We compared the distribution of endogenous LAMP1 in fixed cells and of LAMP1-GFP expressed from a transfection vector in living cells. In accordance with previous reports, we found that the LAMP1-positive membranes appeared associated with intracellular *Salmonella* WT bacteria. In cells infected with the *sifA* mutant strain, most bacteria were not associated with LAMP1 at 10 h p.i. This difference is usually considered as indication for SCV integrity for WT *Salmonella* and SCV loss for *sifA* bacteria. The pattern was similar for endogenous LAMP1 and transfected LAMP1-GFP. However, we observed that the endogenous LAMP1 distribution in fixed cells was mostly patchy and rarely enclosed the bacteria entirely (Fig. 1A). The distribution of LAMP1-GFP was different in living host cells. Here, we observed a complete enclosure with LAMP1-GFP of almost all intracellular WT *Salmonella* (Fig. 1B). Analyses of 3D projections of cells 8 h p.i. with the WT strain indicated that a continuous membrane envelope is formed around the bacteria (S1 Fig., S2 Fig., S2 Movie). The parallel analyses of cells infected with the *sifA* strain showed that many intracellular bacteria completely lost the LAMP1-GFP positive membrane envelope 8 h p.i. (S2 Movie). In the same cell, we also found *sifA Salmonella* that possessed an intact SCV membrane, or that were partially enclosed by LAMP1-GFP positive membranes.

As reported previously [21], we also observed 5 to 10% of infected cells showing massive cytosolic replication of bacteria at early time points p.i. The cytosolic presence is likely to result from high number of invading bacteria and inability of the host cell to maintain SCVs for all internalized bacteria. Based on these observations, we next quantified the SCV integrity in cells infected with *Salmonella* WT and various mutant strains lacking individual SPI2-T3SS effector proteins and compared fixed and live cells. Since the resolution limit of light microscopy did allow sufficiently precise analysis of SCV integrity for larger microcolonies of intracellular bacteria, we focused on clusters containing up to 10 bacteria. Similar to cells infected with *Salmonella* WT, in host cells infected with *sseF*, *sseG*, *sseJ*, *pipB2*, *sopD2* and *sopB* bacteria live cell analyses revealed intact, completely enclosing SCV (Fig. 1C). In cells infected with the *sifA* strain, 45% of the bacteria were completely enclosed by a LAMP1-GFP-positive membrane. In fixed cells, only 57% of intracellular WT bacteria were scored as enclosed by a continuous membrane, and only 10% for the *sifA*-deficient strain.

Taken together, these observations indicate that fixation of *Salmonella*-infected cells results in severe artifacts regarding the appearance of the LAMP1-positive SCV membrane. This finding underlines the requirement for live cell analyses for the study of the intracellular phenotypes of *Salmonella*.

### Systematic analysis of the role of SPI2 effector proteins in dynamics of *Salmonella*-induced filaments

We used our recently established live cell setup [18] to analyze the role of various effector proteins of the SPI2-T3SS for the intracellular fate of *Salmonella* within host cells and the modification of the host endosomal system by *Salmonella* WT and various isogenic mutant strains defective in those effector proteins. Our prior studies indicated that SIF show very dynamic extension and retraction early after the onset of SIF formation, i.e. 4-5 h p.i. of HeLa cells. At later time points, i.e. 8 h p.i. or later, the velocity of extension and retraction of SIF was reduced but the overall number of tubular membrane compartments was increased [18].

**Figure 2:**
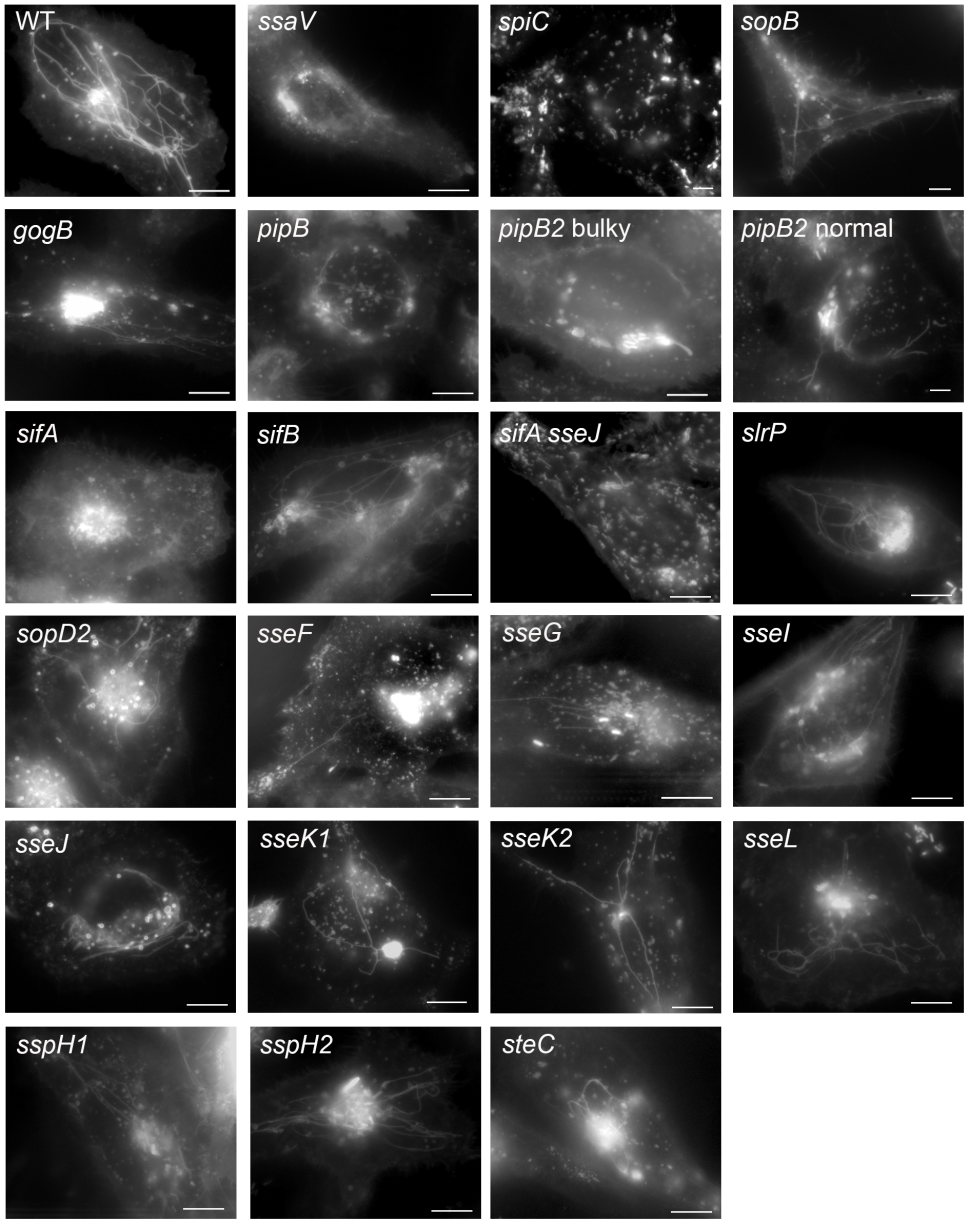
Systematic analyses of role of effector proteins of the SPI2-T3SS for the formation and morphology of tubular endosomes in living host cells. HeLa cells were transiently transfected with a vector for the expression of LAMP1-GFP (white). *Salmonella* WT and a set of isogenic mutant strains with deletions of specific SPI1-T3SS or SPI2-T3SS effector proteins as indicated were used to infect transfected cells. The bacteria harbored plasmids for the constitutive expression of GFP or mCherry. Time lapse series for transfected and infected cells are recorded for the early and late phases of intracellular life, i.e. 4-5 h and 7–9 h post infection (p.i.), respectively. Infection and live cell imaging has been performed independently at least three times per strain, and still images from representative time lapse series are shown. Scale bar, 10 µm. Corresponding movies for each of the experiments shown are available as S3 Movie to S25 Movie.

For a systematic comparative analysis we used time points of 4–5 h and 7–9 h p.i. and imaged the effects of *Salmonella* WT and SPI2-T3SS effector mutants on the endosomal system in HeLa cells transfected with LAMP1-GFP (Fig. 2). A collection of mutant strains was generated that either contained a defect in the SPI2-encoded T3SS or deletions of genes for individual effector proteins of the SPI2-T3SS. Since SopB, an effector translocated by the SPI1-T3SS, has been reported to interfere with the organization of host cell endosomes [22], the phenotype of a *sopB* strain was also investigated. All mutant strains were able to invade HeLa cells and sufficient numbers of LAMP1-GFP-positive infected cells were found for all strains investigated. We observed that the various strains showed rather similar intracellular characteristics during the first phase of intracellular presence. In general, an association of the bacteria with LAMP1-GFP was observed. In accordance with our previous studies, the WT and *ssaV* strains were predominantly found to be enclosed by LAMP1-GFP-positive membranes. Induction of LAMP1-containing membrane tubules was observed in WT-infected cells, but not in cells harboring the *ssaV* strain. The *spiC*-deficient strain was phenotypically indistinguishable from the *ssaV* strain. In cells infected with the *sifA* strain, LAMP1-GFP-positive membranes were found in the vicinity of the bacteria in the early phase of infection (4-5 h p.i.) but at later time points (≥ 8 h p.i.), an association between the intracellular bacteria and LAMP1-postive compartments was usually absent. No formation of LAMP1-positive membrane tubules was observed for cells infected with the *sifA* strain. In cells infected with *sseF* or *sseG* strains, formation of LAMP1-GFP-positive, dynamic tubules was observed, but the fluorescence signal of these compartments was lower than for tubules in WT-infected cells.

We found that other mutant strains defective in one effector showed intracellular characteristics very similar to that of the WT strain. In detail, live cell analyses of cells infected with mutant strains defective in *sifB*, *sseJ*, *sseK1*, *sseK2*, *sseI*, *sseL*, *sspH1*, *sspH2*, *slrP*, *steC*, *gogB* or *pipB* were performed. For each of these strains, the intracellular bacteria were contained in LAMP1-positive compartments. SIF were induced by *sifB*, *sseJ*, *sseK1*, *sseK2*, *sseI*, *sseL*, *sspH1*, *sspH2*, *slrP*, *steC*, *gogB* or *pipB* mutant strains and the morphology of SIF was comparable to SIF induced by WT *Salmonella*. The SIF induced by *sifB*, *sseJ*, *sseK1*, *sseK2*, *sseI*, *sseL*, *sspH1*, *sspH2*, *slrP*, *steC*, *gogB* or *pipB* mutant strains showed highly dynamic properties in the early phase of intracellular life, and less dynamic characteristics in the later stage of infection. Representative stills from time lapse experiments with the various strains are shown in Fig. 2 and individual movies of lapse series for cells infected with WT and the various mutant strains can be found in S3-S25 Movies.

We also carefully checked the phenotypes in living cells of mutant strains that were previously reported to have altered SCV and/or SIF morphologies. We observed altered intracellular phenotypes and morphologies of endosomal membrane aggregations for the *sseF*, *sseG* and *pipB2* mutant strains. The mutant strains will be described in detail below.

A mutant strain lacking SseJ has been reported to cause increased tubulation of endosomal membranes and it was reported that a *sifA sseJ* double mutation can compensate the loss of the SCV observed in the *sifA* strains [23]. Live cell imaging of cells infected with the *sseJ* strain did not reveal detectable differences in the organization and dynamics of SIF. We investigated the characteristics of a mutant strain deficient in both *sifA* and *sseJ*. Similar to the phenotype of the *sifA* strain, we found that the *sifA sseJ* strain did not induce SIF. However, the *sifA sseJ* strain did not lose the association with LAMP1-containing membranes and at 8 h p.i., a majority of *sifA sseJ Salmonella* were contained in LAMP1-GFP-positive continuous compartments. The morphologies and dynamics of SIF in *sopD2*-infected host cells were similar to those of *Salmonella* WT-induced SIF.

### Chemical fixation alters morphology of SIF

We compared the appearance of SIF in living and fixed cells. In living cells infected with *Salmonella* WT, highly dynamic SIF appeared as early as 3.5 h p.i. while more static SIF were detected at time points of 8 h or later p.i. SIF were only rarely detected in fixed cells at the early times points. In cells infected with *sseF* or *sseG* strains, an aberrant SIF phenotype has been described [9]. The discontinuous structures observed in fixed cells infected with *sseF* or *sseG* mutant strains were referred to as pseudo-SIF to distinguish them from the aggregates induced by WT *Salmonella*. Pseudo-SIF appeared as linear arrays of spherical vesicles with a discontinuous staining for LAMP1 and other endosomal membrane markers.

**Figure 3:**
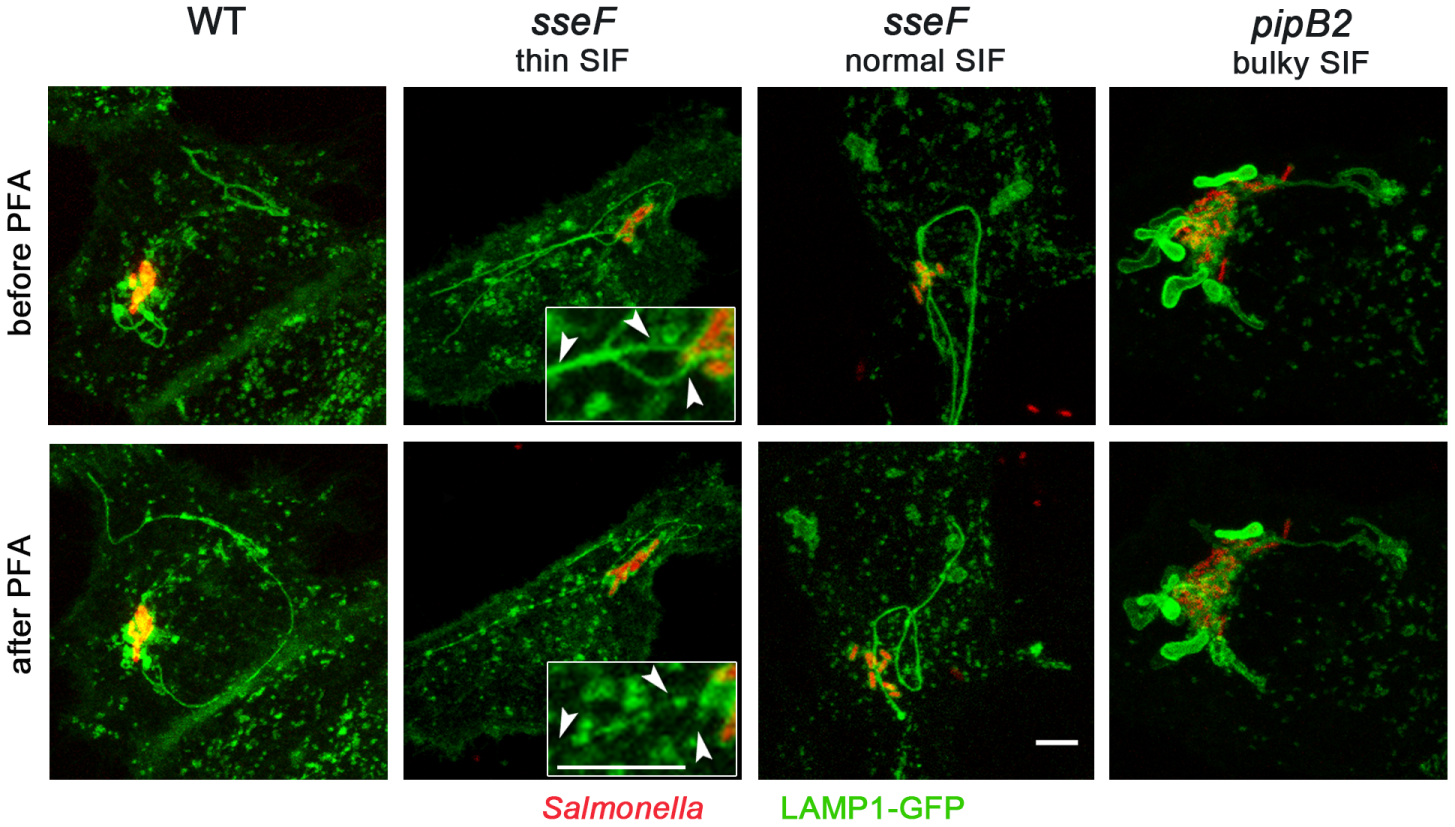
Effect of fixation on morphology of tubular endosomal aggregates. HeLa cells were transfected with LAMP1-GFP (green) and subsequently infected with *Salmonella* WT, *sseF* or *pipB2* strains harboring a plasmid for the expression of mCherry (red). Imaging of infected cells in chamber slides was performed prior fixation and positions were stored for repositioning of a motorized stage. Chamber slides were removed from the stage, cell were washed and fixed by addition of 3% PFA in PBS. Subsequently, the chamber slide was repositioned on the microscopy stage and the positions were relocated in order to image the same cell after fixation. Representative infected cells were imaged at 6-8 h p.i. prior and after fixation. The appearance of tubular structures in an *sseF*-infected cell is shown in detail micrographs and indicated by arrowheads. Scale bars, 10 µm.

Using the live cell setup, we observed a different appearance of SIF in cells infected with *sseF, sseG or pipB2* strains (Fig. 2). We compared the morphology of SIF in *Salmonella*-infected LAMP1-GFP expressing HeLa cells during live cell imaging and after fixation by *para*-formaldehyde (PFA) (Fig. 3). We were able to relocate cells after fixation using a motorized microscope stage. The time interval required for removal of slides, washing and fixation resulted in altered subcellular positions of some of the dynamic SIF, but no gross alteration of SIF morphology was observed for WT-infected cells. After infection with the *sseF* strain, most cells showed dynamic and thin SIF. Imaging of the same cell after fixation with PFA revealed that the thin tubular SIF were destroyed and ‘beads on a string’ appearance of LAMP1-GFP-positive compartments was observed. A low number of *sseF*-infected cells possessed SIF that were indistinguishable from SIF in WT-infected cells. These tubular structures were not fragmented by PFA fixation (data not shown). For SIF induced by the *sseG* mutant we observed the same fixation phenotypes (data not shown). This observation indicates that *sseF*- and *sseG*-induced thin SIF are prone to fragmentation by chemical fixation. For cells infected with the *pipB2* mutant strain we observed bulky, non-dynamic SIF in a fraction of infected cells. The integrity of these bulky, non-dynamic SIF was not affected by PFA fixation (Fig. 3). We also compared PFA fixation to glutaraldehyde (GA) fixation, as a method commonly used for ultrastructural analyses (S3 Fig.). The effect of fixation on *sseF* infected cells was followed for single cells on the microscope stage prior and after addition of fixation agents (S3B Fig.). GA fixation resulted in a lower degree of fragmentation of SIF than PFA. However, GA resulted in increased autofluorescence of host cells, thus interfering with epifluorescence microscopy.

### Time-resolved analysis of endosomal escape of *sifA*-deficient *Salmonella*

**Figure 4:**
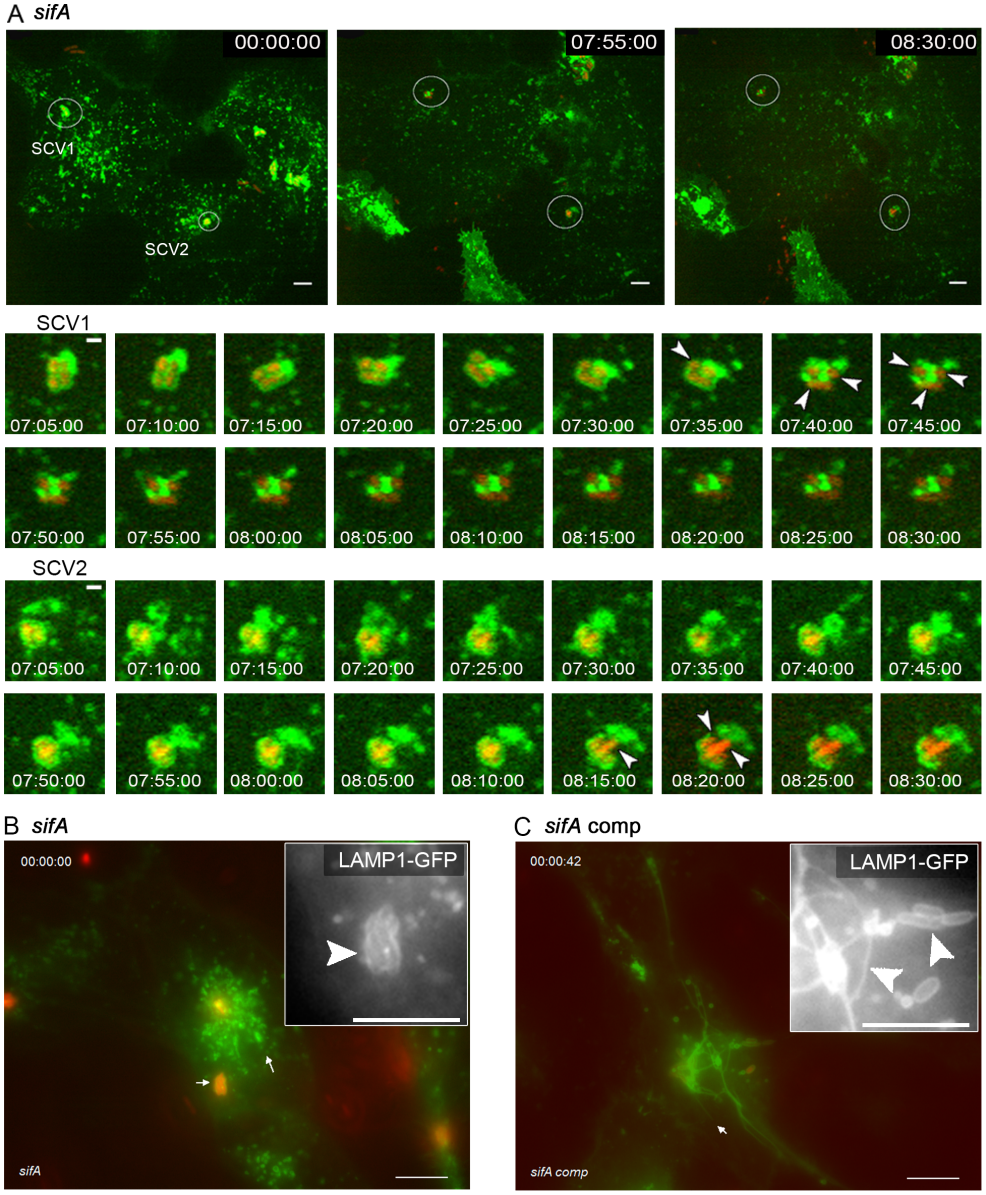
Intracellular fate of the *sifA* strain in living host cells. HeLa cells were transfected with LAMP1-GFP (green) and infected with the *sifA* strain (A, B) or completed *sifA* strain (C) constitutively expressing mCherry (red). A) Live cell imaging of infected cells was performed over 10 h and time lapse series with intervals of 5 min were obtained. The stills correspond to a movie shown as S26 Movie. Detailed micrographs of two clusters of bacteria are shown that lost the integrity of the SCV about 7 h 35 min (SCV1) or 8 h 15 min (SCV2) p.i. B) A *sifA*-infected HeLa cell at 7 h p.i. showing intracellular microcolonies enclosed by a continuous LAMP1-GFP-positive SCV but lacking tubular compartments. C) Restoration of tubular LAMP1-GFP-positive compartments by *sifA* complemented with p*sifA*. Inserts show the GFP channel. The time point of imaging is indicated as hh:mn:ss. Scale bars, 10 µm and 1 µm in overview and detail micrographs in A), respectively; 10 µm in B) and C).

In accordance with previous studies and the initial screen shown in Fig. 2, no induction of SIF was observed in living cells infected with the *sifA* strain. To investigate the fate of the *sifA* strain in more detail, we followed intracellular bacteria over a long time period by live cell imaging (Fig. 4, S26 Movie). Time lapse sequences indicated that *sifA* bacteria were enclosed by LAMP1-GFP-containing membranes for several hours. The bacteria replicated within this compartment and initiated the formation of smaller microcolonies (see example in Fig. 4B). Absence of LAMP1-positive membranes around intracellular *Salmonella* has been considered as indicator for the loss of SCV integrity and release into the cytosol [7]. At late time points after infection, typically 7 to 8 h p.i., microcolonies of *sifA Salmonella* started to lose the continuous LAMP1-GFP-positive compartment (Fig. 4A). These events were rather rapid, and within 20-30 min, the bacteria were completely devoid of an SCV or only co-localized with patches of LAMP1-GFP membranes (Fig. 4A, S26 Movie). Subsequently, *sifA* bacteria continued replication, and the massive cytosolic replication resulted in host cell damage and loss of these cells (not shown). SCV integrity as well as SIF formation was restored in *sifA* mutant strains harboring a plasmid for *sifA* expression (Fig. 4C).

### *sseF*- or *sseG*-deficient *Salmonella* induce thin, dynamic SIF

**Figure 5:**
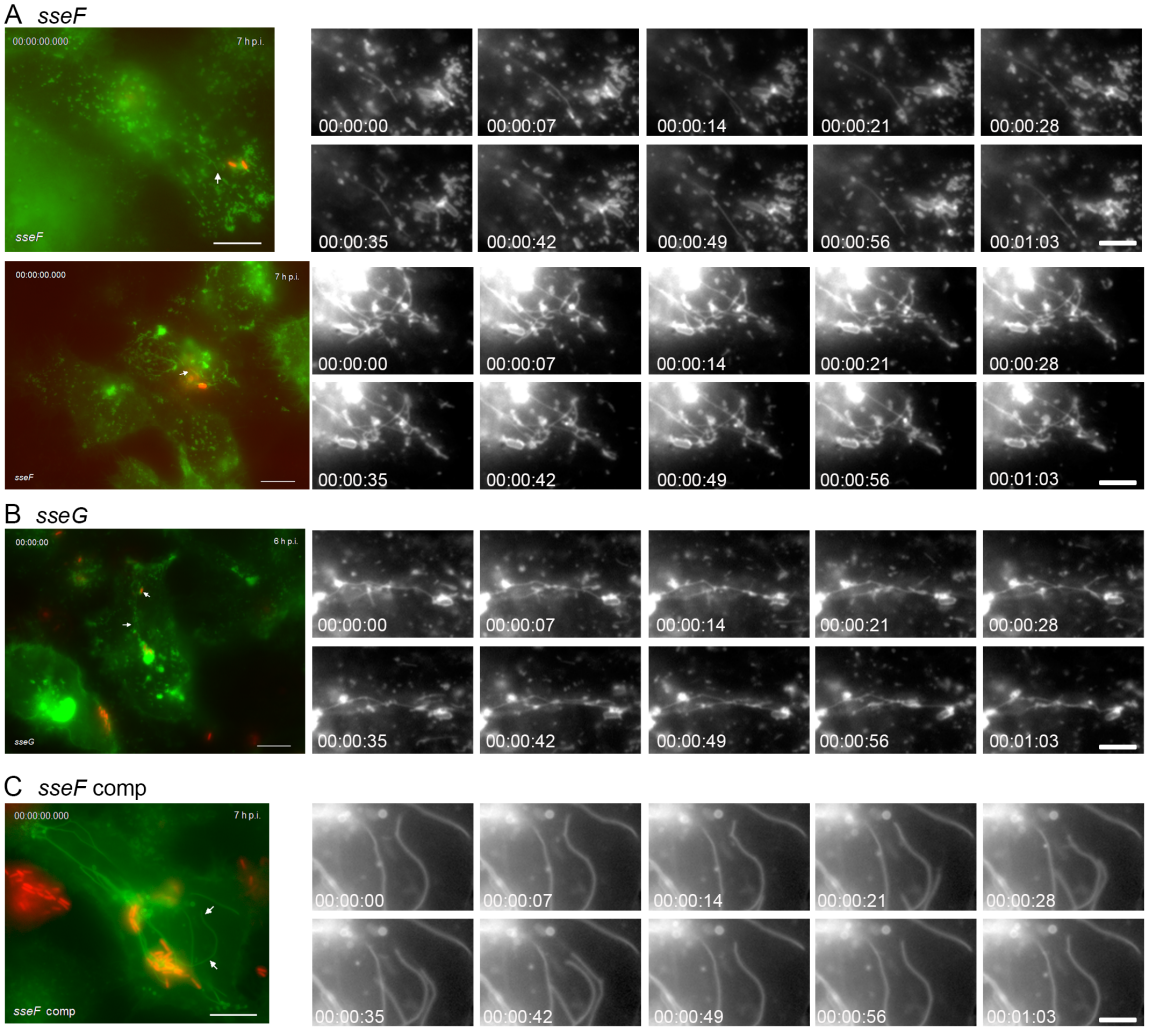
Intracellular fates of *sseF-* or *sseG-*deficient strains in living host cells. Infection was performed as described for Fig. 4 with strains deficient in *sseF* (A), *sseG* (B), or the complemented *sseF* strain (C). Time lapse series were generated at 6 to 7 h p.i. as indicated. Note the appearance of thin, highly dynamic LAMP1-GFP-positive tubules in *sseF*- or *sseG*-infected cells. Scale bars, 10 µm and 2 µm in overview and detail micrographs, respectively. The stills correspond to a movie shown as S27 Movie.

Cells infected with *sseF* or *sseG* mutant strains showed altered SIF morphology and phenotypes induced by both mutant strains were similar. Extensive networks of long, but weakly LAMP1-GFP-positive tubules were observed (Fig. 5). The tubules appeared of thinner diameter and were difficult to visualize due to the lower signal intensity for the membrane marker. As described before (Fig. 3), the tubular structures induced by the *sseF* strain were more sensitive to fragmentation by chemical fixation than SIF in WT-infected cells, suggesting a different organization of the membrane compartments. The tubular membrane compartments in *sseF*- or *sseG*-infected cells were highly dynamic and rapid extension and shrinking of tubules was observed for both *sseF-* or *sseG*-infected cells (Fig. 5A and Fig. 5B, respectively). Complementation by the plasmids expressing *sseF* or *sseG* fully restored the formation of SIF with WT appearance (see Fig. 5C for *sseF* complementation).

### *pipB2*-deficient *Salmonella* induce bulky, non-dynamic SIF

**Figure 6:**
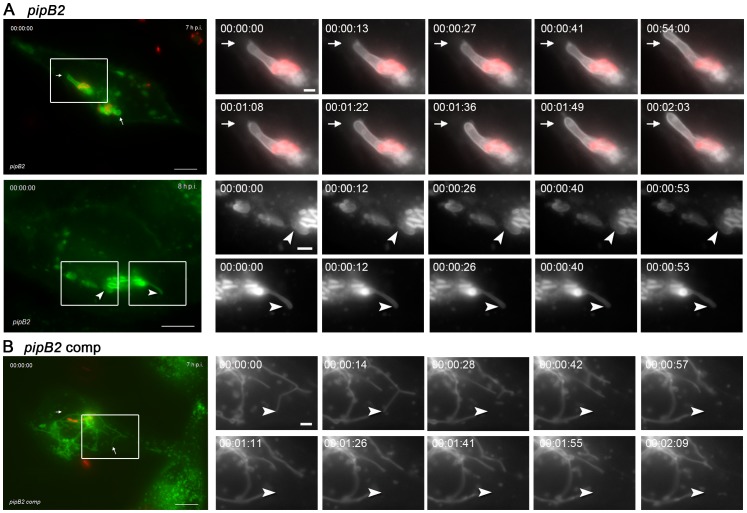
Intracellular fate of the *pipB2* strain in living host cells. The experiment was performed as described for Fig. 4 with a strain deficient in *pipB2* (A), or the complemented *pipB2* strain (B). Time lapse series were generated at 7 or 8 h p.i. as indicated. Note the appearance of bulky, non-dynamic LAMP1-GFP-positive tubules in *pipB2*-infected cells. Scale bars, 10 µm and 2 µm in overview and detail micrographs, respectively. The stills correspond to a time lapse series shown as S28 Movie.

Among the various mutant strains investigated, the most aberrant intracellular phenotype was observed for the *pipB2-*deficient strain. About 30–70% of the *pipB2*-infected host cells per field of view showed SIF that were indistinguishable from SIF in WT-infected cells (see Fig. 2B). However, for the other faction of *pipB2*-infected host cells, we observed extremely large and stumpy LAMP1-GFP-positive membrane compartments that will be referred to as ‘bulky SIF’ (Fig. 3, Fig. 6A). Bulky SIF were generally connected to an SCV. In contrast to SIF with normal appearance induced by the *pipB2* strain, bulky SIF were almost static and the extension or contraction of the compartments was rather slow. The reason for the simultaneous appearance of normal and bulky SIF after infection with *pipB2*-deficient *Salmonella* is unknown so far. Plasmid complementation of the *pipB2* mutant strain fully restored normal SIF formation (Fig. 6B).

### Variant forms of SIF are accessible to endocytic tracers

**Figure 7:**
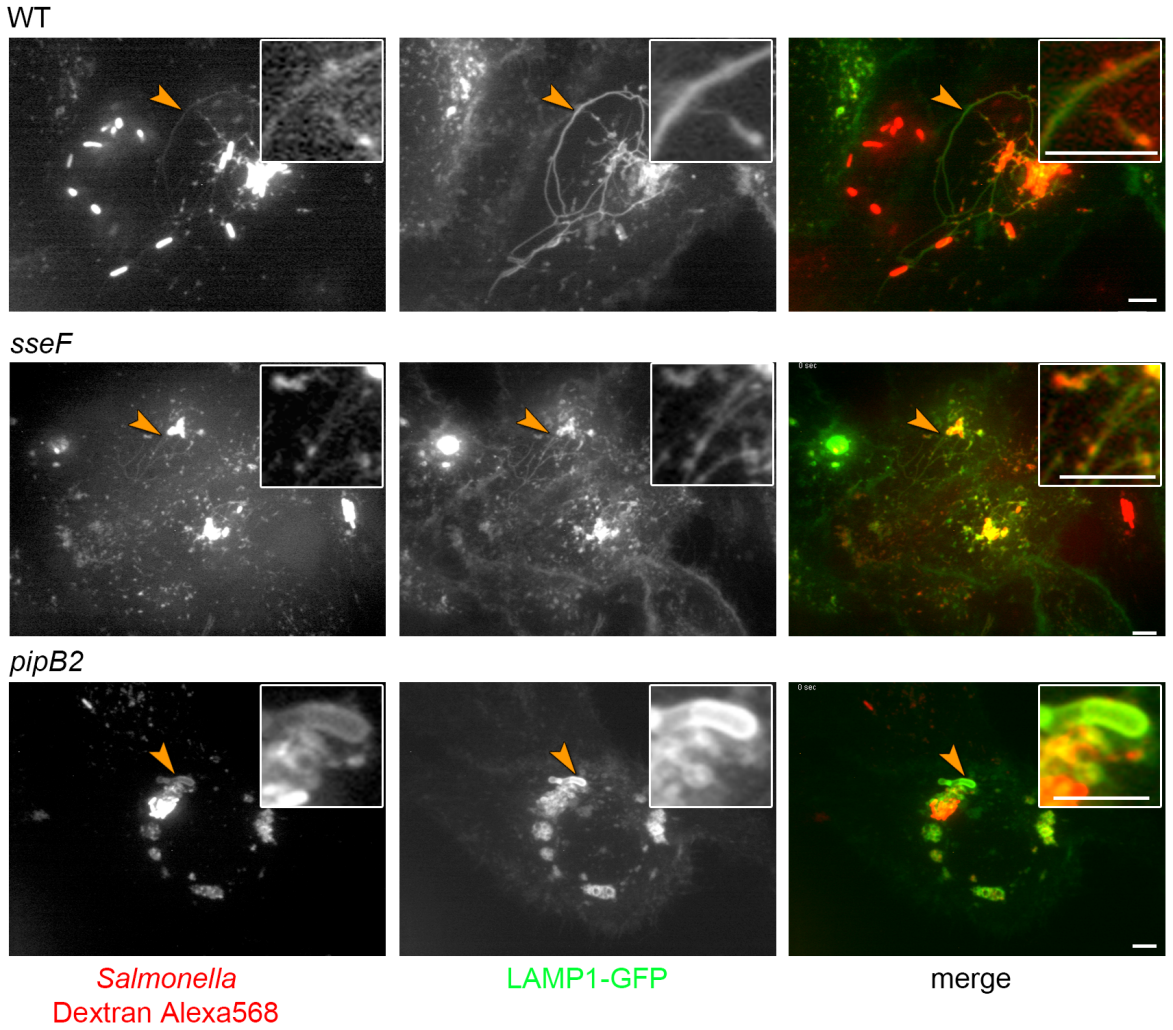
Access of endocytic cargo to SIF. HeLa cells were transfected with a vector for expression of LAMP1-GFP (green) and infected with WT, *sseF* or *pipB2* strains expressing mCherry (red) as indicated. At 4 h p.i., the cells were pulse-chased with Dextran-Alexa568 (red) for 3 h. Live cell imaging was performed at 7–8 h p.i. Note the appearance of dynamic extended tubular structures double positive for LAMP1-GFP and Dextran-Alexa568 in cells infected with WT or *sseF* strains, as well as double positive bulky structures in *pipB2*-infected cells. Scale bars, 2 µm.

We and other previously reported that the SIF lumen is accessible to fluid phase tracers and fluorescent probes such as Dextran-Alexa568 can be used to follow endocytic uptake and interaction with SIF [18], [24]. Here, we followed the interaction of endocytosed material with the LAMP1-GFP-positive membrane compartments induced in cells infected by *Salmonella* WT, *sseF* or *pipB2* strains (Fig. 7). At 7-8 h p.i., cells infected with WT *Salmonella* showed an extensive network of SIF. The majority of SIF also showed the presence of the Dextran tracer in the lumen of the compartment and labeling appeared continuous over the length of the SIF. Presence of Dextran-Alexa568 was also observed for the thin SIF observed in *sseF*-infected cells and the bulky SIF of the *pipB2*-infected cells. The LAMP1-GFP signal of SIF in *sseF*-infected cells was weak, and similar weak signals were observed for luminal tracer in these structures. In contrast, the bulky SIF in cells infected in *pipB2*-deficient *Salmonella* also showed strong Dextran-Alexa568 signals.

The data indicate that although morphologically distinct tubular compartments are induced in *sseF*- or *pipB2*-infected cells, these compartments are in interchange with endocytosed material. The fusogenic properties of these compartments appear similar to the SIF induced by WT *Salmonella*.

### Kinetics of formation of SIF

**Figure 8:**
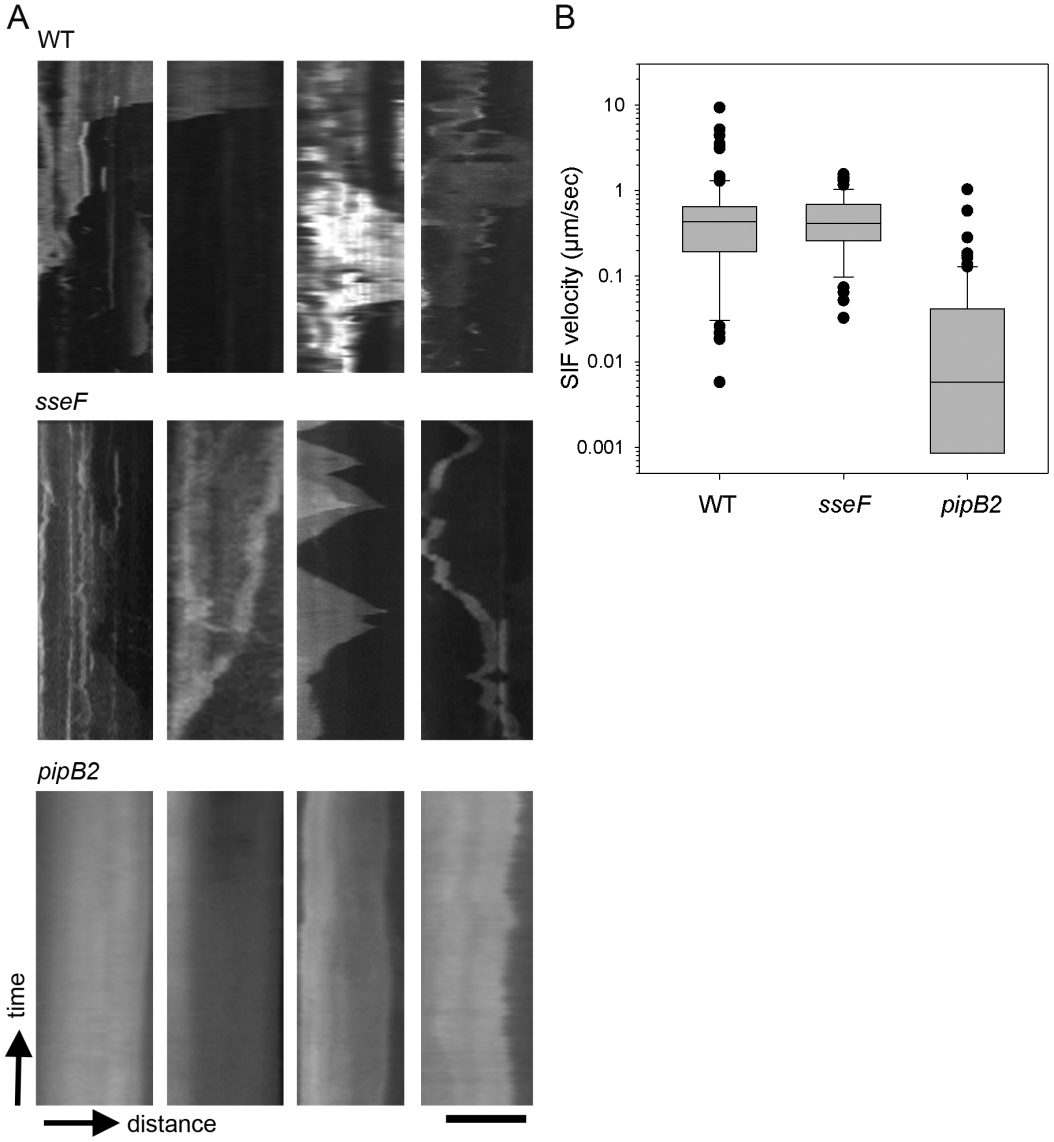
Dynamics of SIF in HeLa cells infected with *Salmonella* WT and various mutant strains. HeLa cells were transfected with the LAMP1-GFP construct and infected with *Salmonella* WT, *sseF*-deficient or *pipB2*-deficient strains. At 4–6 h p.i., time lapse series of infected cells were recorded. SIF were identified and the length of individual SIF was determined in 100 images of the time lapse series, representing 500 msec time delay between each frame. Kymographs SIF were generated using the EMBL Image J software. A) Four representative Kymographs are shown each for cells infected with *Salmonella* WT, *sseF*, or *pipB2* strains as indicated. Scale bar, 5 µm. B) The velocities of SIF extension or collapse were calculated from kymographs generated for cells 5 h p.i., combining 100 events of extension and contraction per strain. The data are displayed as box and whisker plot.

Our previous analyses of the dynamics of SIF in HeLa cells infected with *Salmonella* WT indicated that SIF extended in the early phase of intracellular life [18]. Here we investigated if the velocity of SIF extension and collapse was affected by the function of effector proteins (Fig. 8). The overall mean velocity of SIF in WT-infected cells, inducing events of extension and collapse, was 0.71 µm x sec^−1^ (± 0.12 standard error of mean, SE). The *sifA* mutant strain was not further analyzed since tubular endosomal aggregations were entirely absent. The thin tubular aggregates induced by the *sseF*-deficient strain exhibited only slightly reduced dynamic characteristics, i.e. 0.51 µm x sec^−1^ ± 0.04 SE. The bulky structures induced by the *pipB2* strain showed a certain degree of dynamic movement, but the overall motility of *pipB2* strain-induced bulky SIF was much lower, i.e. 0.05 µm x sec^−1^ ± 0.01 SE.

### Ultrastructural features of SIF induced by WT and various mutant strains

**Figure 9:**
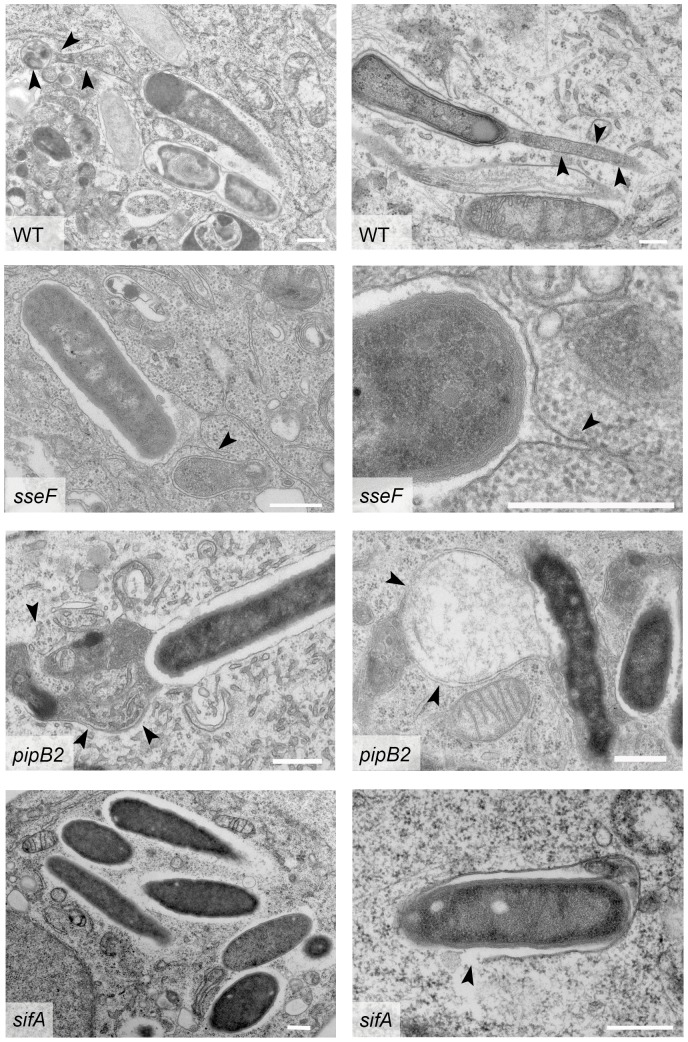
Ultrastructural features of tubular membrane compartments induced by *Salmonella* WT and various mutant strains. HeLa cells were infected with WT *Salmonella* or mutant strains lacking SPI2 effectors as indicated. The cells were processed for TEM analyses at 10 h p.i. Representative cells for each infecting strain are shown. Arrowheads indicate the membranes of tubular compartments. Scale bars, 500 nm.

The phenotypic differences in live cell studies of SIF induced by *Salmonella* WT and mutant strains deficient in *sifA*, *sseF*, *sseG* or *pipB2* also prompted us to investigate the ultrastructure of SIF in infected HeLa cells (Fig. 9). For all strains used, a certain proportion of cytosolic bacteria were identified, in line with common observations in the field [21]. In the following we describe the observations repeatedly made in ultrathin section of WT or mutant strain infected cell.

As reported before [18], intracellular WT bacteria were usually found in SCV with continuous membrane compartments. Extensive tubular membrane compartments were frequently observed in WT-infected cells. However, due to the low volume of a cell accessible by TEM of ultrathin sections, detection of tubular membrane structures connected to intracellular *Salmonella* was rare. However, analyses of a larger number of sections of infected cells identified tubular membrane structures in infected cells. Events of tubular membrane compartments emanating from the SCV and example are shown in Fig. 9. The thin, but continuous structures observed in living cells infected with *sseF* or *sseG* strains were occasionally observed in ultrastructural analyses. A reliable identification of these thin structures was only possible if tubules were associated with SCV and the detection of thin SIF in distal parts was complicated by presence of other tubular organelles such as ER with similar appearance. In *pipB2*-infected cells, the remarkably large and bulky membrane structures were identified and appeared of similar dimensions as estimated from live cell imaging. For cells infected with the *sifA* strain, at 10 h p.i., bacteria were found that were enclosed by a membrane, as well as bacteria that were free in the cytoplasm. As shown in Fig. 9, in some samples intracellular bacteria were only partially associated with a SCV membrane. Interestingly, the bacterial outer membrane was not in direct contact with the host cell cytosol, but completely enclosed by a rim of electron-permissive material. Rarely, *sifA*-deficient bacteria were found that were partially enclosed with a SCV membrane and partially in contact with the host cell cytosol (see Fig. 9, *sifA*, right panel).

## Discussion

The intracellular life of *Salmonella enterica* inside host cells is based on its ability to modify basic host cell functions for the benefit of the pathogen. These modifications result in a highly dynamic interplay of host cell and pathogen. Much of our knowledge on the intracellular phenotypes of *Salmonella* is based on observation of infected host cells after fixation and immuno-staining. However, these approaches are commonly hampered by the fact that the fate of individual host cells and intracellular bacteria cannot be followed over the time course of infection. For example, neither the escape of individual bacteria from the SCV, nor the development and morphologic variation of SIF can be analyzed. Thus, we and others devised experimental setups for live cell imaging that follow the fate of individual bacteria in infected host cells [18], [19], [25]. These studies demonstrated the highly dynamic characteristics of SIF and other filamentous membrane structures and allowed the quantification of velocities for growth and contraction. Our work reveals two major differences due to fixation of cells. i) Intracellular *Salmonella* are enclosed by continuous lgp-positive compartments and the integrity of the SCV is lost in fixed cells. ii) Fixation also results in loss of a portion of the tubular structures, as observed for cells infected with *Salmonella* lacking SseF or SseG. We propose that fixed cell analyses only detect a subset of tubular membrane compartments, while other, more fragile species of tubules are destroyed by fixation. Previous studies indicated that fixation has dramatic effects on the integrity of endosomal membrane and may lead to the loss of vesicular content [26].

We deployed the live cell setup to investigate the roles of individual effector proteins on SIF formation in living host cells and to determine the effect of effector proteins in the dynamics of SIF. Our data demonstrate that the dynamic properties of SIF in living cells are affected most dramatically by defects in SifA and PipB2, and to a lesser extent by defects in SseF or SseG. *sifA*-infected cells lacked LAMP1-positive dynamic tubular membrane aggregations and loss of SCV started 7–9 h p.i., resulting in more than 50% of bacteria lacking SCV at 16 h p.i. A limitation of our analysis obviously is the low number of cells that can be analyzed for intracellular phenotypes and SIF dynamics. Furthermore, the temporal resolution for the events of phagosomal escape is limited by the fact that the time point of escape can vary within a range of two hours.

In cells infected with *sseF*- or *sseG*-deficient strains, the formation of thin tubular structures was observed. These structures had dynamic properties similar to the SIF observed in *Salmonella* WT-infected cells. For cells infected with *sseF*- or *sseG*-deficient strains, the lack of SIF formation was reported [27]. However, subsequent work described ‘pseudo-SIF’ [9] in cells infected with *sseF* or *sseG* mutant strains, and pseudo-SIF are considered as discontinuous tubular aggregations. We could demonstrate that the discontinuous appearance of pseudo-SIF is the result of chemical fixation. The continuous, thin tubular extension in living cells disintegrated into pseudo-SIF upon fixation with PFA. Fragmentation of tubular membrane compartments due to PFA fixation has been observed for other cellular compartments [28]. The differences in membrane tension due to various degrees of tubulation may explain the different susceptibilities to PFA-induced fragmentation. The characteristics of filamentous endosomal structures in WT and *sseF*/*sseG* mutant strains may indicate that the existence of distinct populations of *Salmonella*-induced tubular membranes, i.e. fixation-labile and fixation-resistant tubules. Both types of tubules may occur in WT-infected host cells, but only a subset of tubules is detectable after fixation.

A surprising SIF phenotype was observed for a mutant strain deficient in PipB2. In a proportion of infected host cells, large vesicular structures were observed that lacked the dynamic properties of the SIF in WT-infected cells. At present, we cannot explain why cells show either normal SIF or bulky SIF after infection with the *pipB2* mutant strain. One may speculate that the cell cycle status of the host cell may influence the cytoskeletal architecture and dynamics and thereby SIF extension. Furthermore, the amount of the remaining effector proteins that are translocated by the *pipB2* strain is likely to be variable between individual host cells. The bulky appearance of membrane aggregations in *pipB2*-infected cells could be correlated to globular membrane vesicles with diameters of several micrometers. The function of PipB2 as a linker for kinesin was reported by Henry et al. [13], and previous work [12] demonstrated reduced centrifugal extension of SIF in cells infected with *pipB2*-deficient *Salmonella.* Our live cell analyses are in line with a reduced peripheral extension of SIF in the absence of PipB2. Due to the lack of linking of endosomes to the anterograde transport activity of kinesin, large non-dynamic SIF remain in the vicinity of the SCV. Despite the dramatic alteration of SIF structure and dynamics of the *pipB2* mutant strain, the effect on virulence of *Salmonella* is rather small [29]. The centrifugal extension of SIF may not be required to maintain the SCV and to provide membrane material to SCV extension. Our previous work identified SseF, SseG and PipB2 as members of a subset of SPI2-T3SS effectors involved in inhibition of antigen presentation in *Salmonella*-infected dendritic cells [30]. Future work has to reveal if cellular functions involving endosome tubulation, for example transport of MHCII complexes in antigen presenting cells, are affected by PipB2.

The phenotypic differences of LAMP1-positive membrane aggregations in living infected cells were also correlated to changes in the ultrastructural appearance of SIF and the SCV. While SIF induced by *Salmonella* WT commonly showed average diameters of 160-220 nm, the tubular structures induced in *sseF*- or *sseG*-infected cells had diameters of less than 100 nm. Large globular membrane compartments were found in *pipB2*-infected cells and diameters of more than 500 nm were observed.

For the various other effector proteins of the SPI2-T3SS, we did not observe reoccurring alterations in the morphology of SIF in living cells, nor of their frequency, extend or dynamic properties. The role of the effectors in SIF formation was in close correlation with their role in intracellular replication of *Salmonella* in host cells, and *Salmonella* virulence in murine models of infection. Mutant strains defective in *sifA* were most severely attenuated in intracellular replication as well as in systemic virulence, while mutant strains lacking either SseF or SseG showed a moderate reduction of intracellular replication and systemic virulence in the mouse model. In contrast, the rather dramatic phenotype of the *pipB2* strain in living host cells is in contrast to the in vivo phenotype that indicated WT characteristics with respect to systemic virulence.

Endosome tubulation is a common cellular function and has been studied in detail for the tubular network of recycling endosomes in phagocytic cells (reviewed in [31]). The induction of SIF in *Salmonella*-infected cells is a distinct mechanism, resulting ultimately in the conversion of the majority of the late endosomal/lysosomal membrane compartments into a rather static network [18]. Pulse chase experiments with fluid tracers also demonstrate the interconnected nature of the SIF network and the accessibility of various types of endocytic cargo [19], [32]. The understanding of the molecular mechanisms resulting in the formation of these pathogen-induced alterations of the host endosomal system clear requires further investigations, and live cell imaging with the highest temporal and spatial resolution will be instrumental for these studies.

## Materials and Methods

### Bacterial strains and growth condition

*Salmonella enterica* serovar Typhimurium NCTC12023 was used as wild-type strain and all mutant strains were isogenic to this strain. For live cell imaging experiments using spinning disk microscopy, mutant alleles were transduced using P22 transduction into strain background LT2a. We have previously compared intracellular phenotypes of wild-type strains NCTC12023 and LT2a and did not observe differences in the induction of SIF [18]. The construction of mutant strains defective in single effectors has been reported before and the characteristics of the strains are listed in Table 1. Bacterial strains were grown in LB broth or on LB agar containing antibiotics 50 µg x ml^−1^ carbenicillin, 50 µg x ml^−1^ kanamycin or 12 µg x ml^−1^ chloramphenicol if required for the section of plasmids or mutation markers.

**Table 1:** *Salmonella enterica* serovar Typhimurium strains used in this study.

Strains	Genotype, relevant characteristics	Reference
NCTC 12023	Wild type	lab collection
LT2A	attenuated strain, *rpoS*	lab collection
HH107	Δ*sseF*::*aph*	[8]
HH108	Δ*sseG*::*aph*	[8]
EG10128	Δ*spiC*::*aph*	[37]
MvP103	Δ*sseC*::*aph*	[38]
MvP374	Δ*sifB*::*aph*	[34]
MvP375	Δ*sseI*::*aph*	[34]
MvP376	Δ*sspH1*::*aph*	[34]
MvP377	Δs*seJ*::*aph*	[34]
MvP378	Δ*sspH2*::*aph*	[34]
MvP379	Δ*slrP*::*aph*	[34]
MvP392	Δs*seJ*::FRT	this study
MvP450	Δ*sseJ*::FRT *sifA*::mTn*5*	this study
MvP498	Δ*pipB2*::*aph*	[30]
MvP505	Δ*sopD2*::*aph*	[30]
MvP509	Δ*sifA*::*aph*	[30]
MvP570	Δ*sseK1::aph*	[34]
MvP571	Δ*sseK2*::*aph*	[34]
MvP741	Δ*steC*::*aph*	this study
MvP873	Δ*gogB*::FRT	[30]
MvP874	Δ*pipB*::FRT	[34]
MvP1208	Δ*sopB*::*aph*	this study
MvP1033	Δ*sseL*::*aph*	this study

### Generation of mutant strains and plasmid

Further strains defective in genes encoding effector proteins if the SPI1- or SPI2-T3SS were generated by the ‘one step inactivation’ approach basically as described before [33], [34]. If indicated, the *aph* resistance cassette was removed by FLP-mediated recombination, resulting in a remaining FRT scar.

Plasmids used in this study are listed in Table 2. For complementation of mutations in genes encoding effector proteins, low copy number vectors based on pWSK29 are constructed for the expression of the effector proteins under control of their own promoter. To visualize bacteria for live cell imaging, plasmids pFPV25.1 or pFPVmCherry/2 were used. For live cell imaging of plasmid complemented mutant strains, plasmid p3589 was used. p3589 was generated by cloning of the *Pml*I/*Hin*dIII fragment of pFPVmCherry/2 into single copy number vector pETcoco-1. The resulting plasmid confers chloramphenicol resistance and constitutive mCherry expression and is compatible with pWSK29-based plasmids.

**Table 2:** Plasmids used in this study.

Plasmids designation	relevant characteristics	Source reference
pLAMP1-GFP	transfection vector for LAMP1-GFP	[18]
pFPV25.1	const. eGFP expression	[39]
pFPVmCherry/2	const. mCherry expression	[40]
pWSK29	low copy number vector, Amp^R^	[41]
pETcoco-1	single copy number vector, Cm^R^	[42]
p2643	pWSK29 *P_sseA_ sscB sseF*::HA	[43]
p2644	pWSK29 *P_sseA_ sscB sseFG*::HA	[43]
p2104	pWSK29 *P_sifA_ sifA*::M45	[44]
p2621	pWSK29 *P_pipB2_ pipB2*::M45	[29]
p3589	pETcoco-1 const. mCherry	this study

### Cell culture and infection experiments

Human epithelial cell line HeLa (ATCC # CCL-2) was cultured in DMEM with 10% FCS, at 37°C in a humidified atmosphere containing 5% CO_2_. HeLa cells (about 2 × 10^4^ cells) were seeded in wells of an 8 well chamber glass slide (Nunc-LabTek) and allowed to adhere overnight.

For transfection by the calcium phosphate method [35], about 500 µg of plasmid DNA (LAMP1-GFP for single transfections) were mixed with the transfection reagent and added to cells in 8 well chamber slides with DMEM with 10% FCS. Cells were incubated for 4 to 5 h, then medium was changed and fresh DMEM with 10% FCS. Cells were used for infection studies 16 to 18 h after transfection.

For infection, *S.* Typhimurium was sub-cultured from overnight cultures for 3.5 h and the culture was diluted to an OD_600_ of 0.2. Cells were infected at a multiplicity of infection (MOI) of 50 and infection was allowed for a period of 30 min. Subsequently, non-internalized bacteria were removed by washing thrice with PBS and then DMEM containing 10% FCS and gentamicin at 100 µg x ml^−1^ was added to kill remaining extracellular bacteria. After incubation for 1 h, the cell were washed again and phenol red-free, HEPES-buffered imaging medium (MEM, Biochrom) containing 10 µg x ml^-1^ gentamicin was used for the rest of the infection. The chamber slide was then taken for imaging at required time points post infection (p.i.). The chamber slide was mounted on the microscope stage equipped with an incubation chamber maintaining 37°C and 5% CO_2_.

### Fluid phase marker and pulse chase

For tracing the endocytic pathway, fluid phase markers were used. HeLa cells were transfected with LAMP1-GFP, infected as described and 4 h p.i. cells were incubated with 100 µg x ml^−1^ AlexaFluor 568-conjugated dextran, (molecular weight 10,000, Molecular Probes) for 3 h, washed, and incubated for the rest of the experiment with dextran-free medium. Later cells were processed for imaging.

### Fixation and immunostaining

*para*-formaldehyde (PFA) fixation solution was prepared by mixing 30 g PFA with 100 ml pre-heated water in an Erlenmeyer flask. Three drops of 5 M NaOH were added and the mixture was incubated at 60°C with stirring for 15 min., or until fully dissolved. After cooling to room temperature, three drops of 5 M HCl was added and the appropriate amount of 10 × PBS and water were added to obtain 3% PFA in PBS. If required, the pH was adjusted to 7.2 and the solution was stored in aliquots at −20°C.

For immunostaining of fixed cells, HeLa cells (non-transfected) were infected with various *Salmonella* strains as described above and at indicated time points p.i., cells were fixed with 3% PFA for 10 min., washed with PBS and incubated in blocking solution (PBS containing 2% goat serum, 2% bovine serum albumin, 0.1% saponin freshly added from 3% stock solution in PBS). Permeabilized cells were stained with the primary antibodies mouse anti-human LAMP1 (1∶250 in blocking solution) and rabbit anti-*Salmonella* O 4,5 antigen (1∶500) for 1 h, washed with thrice PBS and secondary staining was performed using goat anti-mouse conjugated to Cy3 and goat anti-rabbit conjugated to Alexa568 for 45 min. For glutaraldehyde (GA) fixation, cells were fixed directly on stage by addition of pre-warmed 2.5% GA (Electron Microscopy Sciences) in HEPES buffer (0.2 M HEPES, pH 7.4, 5 mM CaCl_2)_. After fixation for 1 h at 37°C, cells were washed several times with HEPES buffer, and remaining GA was blocked by 50 mM glycine in HEPES buffer for 15 min, followed by washing with buffer.

### Microscopy and imaging

Live cell imaging was performed basically as described before [18]. Imaging studies were done using the Perkin Elmer spinning disc confocal microscope (UltraVIEW-ERS) mounted on a Zeiss Axiovert 200 microscope with an acoustic optical tunable filter (AOTF) for wavelength selection for 4D image acquisitions. The microscope is equipped with a highly sensitive CCD camera, and 5 laser lines along with Nipkow spinning disc for high temporal acquisition with stacks or 4D imaging. Image acquisition was performed using a 100 × Plan Neofluar objective at various time points p.i. and images were stored in a given data format. Live imaging at lower temporal resolution was performed using the Zeiss Axiovert 200M microscope equipped with wide field illumination and an AxioCam MR camera. The resulting movie series were corrected for background fluorescence and bleaching using bleach correction and background subtraction macros available at EMBL Image J (http://www.embl.de/almf/almf_services/services/downloads/index.html). Calculations of the velocity of SIF growth and collapse, and velocities of LAMP1-positive vesicles were done in EMBL Image J using the macro Kymograph written by Arne Seitz [36], or by manual tracking.

For long time lapse series, HeLa cells were transfected and infected with WT, *sseF*, *sifA* or *pipB2* strains expressing GFP or mCherry as described above and after 2 h p.i., the cells were taken for imaging with the Perkin Elmer RS. The time lapse parameters include taking every image between 3 min interval for total time duration of 10 h.

### Electron microscopy

Ultrastructural analyses of HeLa cells infected with various *Salmonella* strains were basically performed as described before [18].

## Supporting Information

S1 Fig Live cell analyses of SCV integrity. HeLa cells expressing LAMP1-GFP (green) were infected with *Salmonella* WT and various mutant strains, each constitutively expressing mCherry (red). Live cell imaging was performed 8 h p.i. Scale bar, 10 µm and 2 µm in overview and detail micrographs, respectively.
(TIFF)

S2 Fig Live cell analyses of SCV integrity. Z sections are shown for representative WT or *sifA*-infected cells from the experiment shown in Figure 1. Scale bar, 10 µm and 2 µm in overview and detail micrographs, respectively.
(TIFF)

S3 Fig Effect of PFA and GA fixation on integrity of SIF. HeLa cells transfected with LAMP1-GFP (green) were infected with *Salmonella sseF* mutant constitutively expressing mCherry (red). At 2-5 h p.i. cells were pulse-chased with the fluid phase marker BSA-Rhodamine (red) for an additional labeling of SCV and SIF. Cells were washed at 8 h p.i. and fixed with 3% PFA (A) or 2.5% GA (B). Representative infected cells with pseudo-SIF phenotypes are shown. C), D) Live cell imaging of infected cells by CLSM was performed at 8 h p.i. Equal amounts of medium containing double concentration of PFA (C) or GA (D) were added to the cells directly on the microscope stage. The effect of fixation was monitored 30 sec and 10 min after addition. Note the vesiculation of membrane tubules after the addition of PFA, resulting in pseudo-SIF formation. GA fixation preserves the morphology of membrane tubules, but induces strong red autofluorescence. Scale bars, 10 µm.
(TIF)

S1 Movie Corresponding to Fig. 1: 3D projection of *Salmonella* (red) WT-infected HeLa cell expressing LAMP1-GFP (green). Live cell imaging performed at 8 h p.i.
(MPG)

S2 Movie Corresponding to Fig. 1: 3D projection of *Salmonella* (red) *sifA* mutant strain-infected HeLa cell expressing LAMP1-GFP (green). Live cell imaging performed at 8 h p.i.
(MPG)

S3 Movie Corresponding to Fig. 2: The movie shows LAMP1-GFP expressing HeLa cells after infection with *Salmonella* WT. Time stamp (upper left corner) indicates hh:mm:ss:ms and the image series was recorded at 8 h p.i. Scale bar, 10 µm.
(MOV)

S4 Movie Corresponding to Fig. 2: The movie shows LAMP1-GFP expressing HeLa cells after infection with *Salmonella ssaV* strain. Time stamp (upper left corner) indicates hh:mm:ss:ms and the image series was recorded at 7 h p.i. Scale bar, 10 µm.
(MOV)

S5 Movie Corresponding to Fig. 2: The movie shows LAMP1-GFP expressing HeLa cells after infection with *Salmonella spiC* strain. Time stamp (upper left corner) indicates hh:mm:ss:ms and the image series was recorded at 7 h p.i. Scale bar, 10 µm.
(MOV)

S6 Movie Corresponding to Fig. 2: The movie shows LAMP1-GFP expressing HeLa cells after infection with *Salmonella sopB* strain. Time stamp (upper left corner) indicates hh:mm:ss:ms and the image series was recorded at 6 h p.i. Scale bar, 10 µm.
(MOV)

S7 Movie Corresponding to Fig. 2: The movie shows LAMP1-GFP expressing HeLa cells after infection with *Salmonella gogB* strain. Time stamp (upper left corner) indicates hh:mm:ss:ms and the image series was recorded at 7 h p.i. Scale bar, 10 µm.
(MOV)

S8 Movie Corresponding to Fig. 2: The movie shows LAMP1-GFP expressing HeLa cells after infection with *Salmonella pipB* strain. Time stamp (upper left corner) indicates hh:mm:ss:ms and the image series was recorded at 6 p.i. Scale bar, 10 µm.
(MOV)

S9 Movie Corresponding to Fig. 2: The movie shows LAMP1-GFP expressing HeLa cells after infection with *Salmonella pipB2* strain. For cells infected with the *pipB2* mutant strain, either SIF of normal appearance or bulky LAMP1-GFP-positive compartments were observed. This example shows the bulky SIF phenotype. Time stamp (upper left corner) indicates hh:mm:ss:ms and the image series was recorded at 7 h p.i. Scale bar, 10 µm.
(MOV)

S10 Movie Corresponding to Fig. 2: The movie shows LAMP1-GFP expressing HeLa cells after infection with *Salmonella pipB2* strain. For cells infected with the *pipB2* mutant strain, either SIF of normal appearance or bulky LAMP1-GFP-positive compartments were observed. This example shows the normal SIF phenotype. Time stamp (upper left corner) indicates hh:mm:ss:ms and the image series was recorded at 7 h p.i. Scale bar, 10 µm.
(MOV)

S11 Movie Corresponding to Fig. 2: The movie shows LAMP1-GFP expressing HeLa cells after infection with *Salmonella sifA* strain. Time stamp (upper left corner) indicates hh:mm:ss:ms and the image series was recorded at 7 h p.i. Scale bar, 10 µm.
(MOV)

S12 Movie Corresponding to Fig. 2: The movie shows LAMP1-GFP expressing HeLa cells after infection with *Salmonella sifB* strain. Time stamp (upper left corner) indicates hh:mm:ss:ms and the image series was recorded at 7 h p.i. Scale bar, 10 µm.
(MOV)

S13 Movie Corresponding to Fig. 2: The movie shows LAMP1-GFP expressing HeLa cells after infection with *Salmonella sifA sseJ* double mutant strain. Time stamp (upper left corner) indicates hh:mm:ss:ms and the image series was recorded at 8 h p.i. Scale bar, 10 µm.
(MOV)

S14 Movie Corresponding to Fig. 2: The movie shows LAMP1-GFP expressing HeLa cells after infection with *Salmonella slrP* strain. Time stamp (upper left corner) indicates hh:mm:ss:ms and the image series was recorded at 8 h p.i. Scale bar, 10 µm.
(MOV)

S15 Movie Corresponding to Fig. 2: The movie shows LAMP1-GFP expressing HeLa cells after infection with *Salmonella sopD2* strain. Time stamp (upper left corner) indicates hh:mm:ss:ms and the image series was recorded at 7 h p.i. Scale bar, 10 µm.
(MOV)

S16 Movie Corresponding to Fig. 2: The movie shows LAMP1-GFP expressing HeLa cells after infection with *Salmonella sseF* strain. Time stamp (upper left corner) indicates hh:mm:ss:ms and the image series was recorded at 7 h p.i. Scale bar, 10 µm.
(MOV)

S17 Movie Corresponding to Fig. 2: The movie shows LAMP1-GFP expressing HeLa cells after infection with *Salmonella sseG* strain. Time stamp (upper left corner) indicates hh:mm:ss:ms and the image series was recorded at 7 h p.i. Scale bar, 10 µm.
(MOV)

S18 Movie Corresponding to Fig. 2: The movie shows LAMP1-GFP expressing HeLa cells after infection with *Salmonella sseI* strain. Time stamp (upper left corner) indicates hh:mm:ss:ms and the image series was recorded at 8 h p.i. Scale bar, 10 µm.
(MOV)

S19 Movie Corresponding to Fig. 2: The movie shows LAMP1-GFP expressing HeLa cells after infection with *Salmonella sseJ* strain. Time stamp (upper left corner) indicates hh:mm:ss:ms and the image series was recorded at 7 h p.i. Scale bar, 10 µm.
(MOV)

S20 Movie Corresponding to Fig. 2: The movie shows LAMP1-GFP expressing HeLa cells after infection with *Salmonella sseK1* strain. Time stamp (upper left corner) indicates hh:mm:ss:ms and the image series was recorded at 7 h p.i. Scale bar, 10 µm.
(MOV)

S21 Movie Corresponding to Fig. 2: The movie shows LAMP1-GFP expressing HeLa cells after infection with *Salmonella sseK2* strain. Time stamp (upper left corner) indicates hh:mm:ss:ms and the image series was recorded at 7 h p.i. Scale bar, 10 µm.
(MOV)

S22 Movie Corresponding to Fig. 2: The movie shows LAMP1-GFP expressing HeLa cells after infection with *Salmonella sseL* strain. Time stamp (upper left corner) indicates hh:mm:ss:ms and the image series was recorded at 6 h p.i. Scale bar, 10 µm.
(MOV)

S23 Movie Corresponding to Fig. 2: The movie shows LAMP1-GFP expressing HeLa cells after infection with *Salmonella sspH1* strain. Time stamp (upper left corner) indicates hh:mm:ss:ms and the image series was recorded at 8 h p.i. Scale bar, 10 µm.
(MOV)

S24 Movie Corresponding to Fig. 2: The movie shows LAMP1-GFP expressing HeLa cells after infection with *Salmonella sspH2* strain. Time stamp (upper left corner) indicates hh:mm:ss:ms and the image series was recorded at 8 h p.i. Scale bar, 10 µm.
(MOV)

S25 Movie Corresponding to Fig. 2: The movie shows LAMP1-GFP expressing HeLa cells after infection with *Salmonella steC* strain. Time stamp (upper left corner) indicates hh:mm:ss:ms and the image series was recorded at 6 h p.i. Scale bar, 10 µm.
(MOV)

S26 Movie Corresponding to Fig. 4: A time lapse series of 1 to 10 h p.i. was recorded for LAMP1-GFP-expressing HeLa cells infected with the *sifA* mutant strain. Note: at the bottom of the movie appear the highlighted SCVs that loose the LAMP1-GFP marker. Scale bar, 5 µm.
(MOV)

S27 Movie Corresponding to Fig. 5: The movie shows LAMP1-GFP expressing HeLa cells infected by the *sseF* strain (*sseF*) or the complemented *sseF* strain (*sseF* comp). Time lapse series were recorded 7 h p.i. and annotations are as described for Movie S3. Scale bar, 10 µm.
(MOV)

S28 Movie Corresponding to Fig. 6: The movie shows LAMP1-GFP expressing HeLa cells infected by the *pipB2* strain (*pipB2*) or the complemented *pipB2* strain (*pipB2* comp). Time lapse series were recorded at 7 h or 8 h p.i. as indicated and annotations are as described for Movie S3. Scale bars, 10 µm in overview and 2 µm in detail.
(MOV)
