# Supplementary figures and images for: Live Cell Imaging Reveals Novel Functions of Salmonella enterica SPI2-T3SS Effector Proteins in Remodeling of the Host Cell Endosomal System
Source: PLoS One. 2014 Dec 18;9(12):e115423. doi: 10.1371/journal.pone.0115423 (PMC4270777; doi:10.1371/journal.pone.0115423)

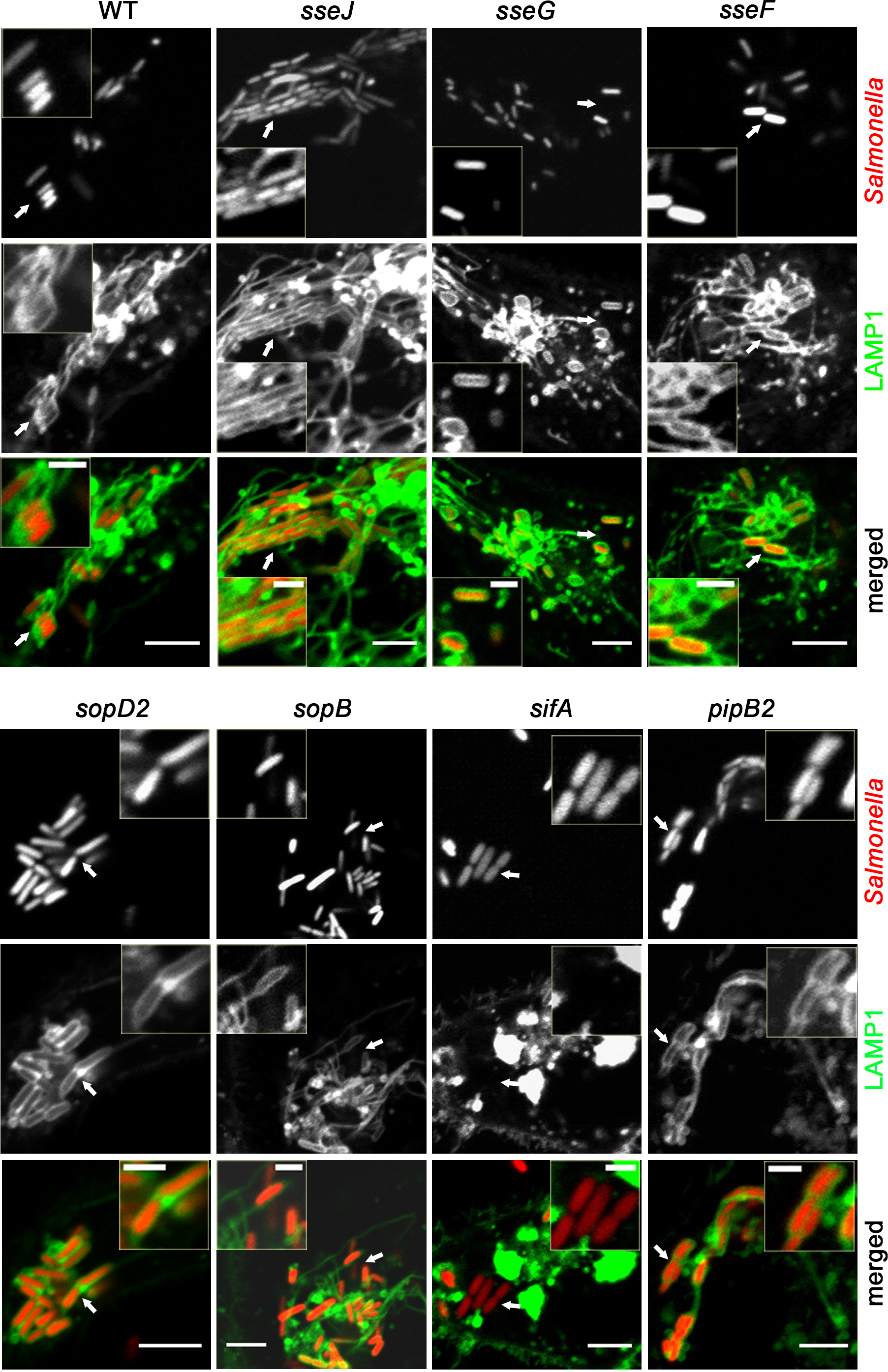

Supplement: S1 Fig — Live cell analyses of SCV integrity. HeLa cells expressing LAMP1-GFP (green) were infected with Salmonella WT and various mutant strains, each constitutively expressing mCherry (red). Live cell imaging was performed 8 h p.i. Scale bar, 10 µm and 2 µm in overview and detail micrographs, respectively. (TIFF) [file pone.0115423.s001.tiff]

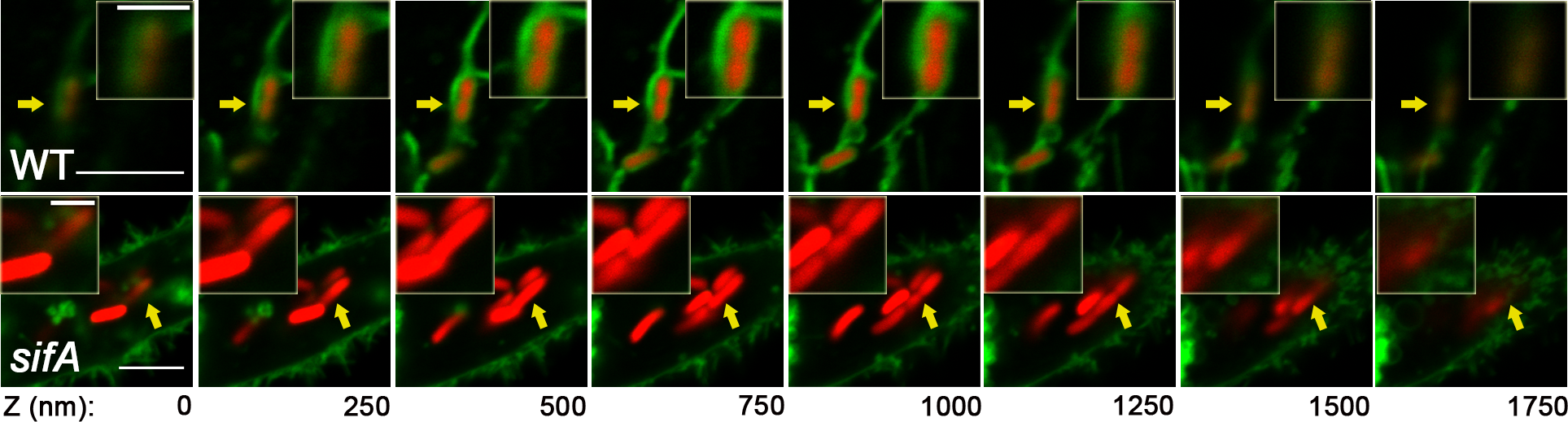

Supplement: S2 Fig — Live cell analyses of SCV integrity. Z sections are shown for representative WT or sifA-infected cells from the experiment shown in Figure 1. Scale bar, 10 µm and 2 µm in overview and detail micrographs, respectively. (TIFF) [file pone.0115423.s002.tiff]

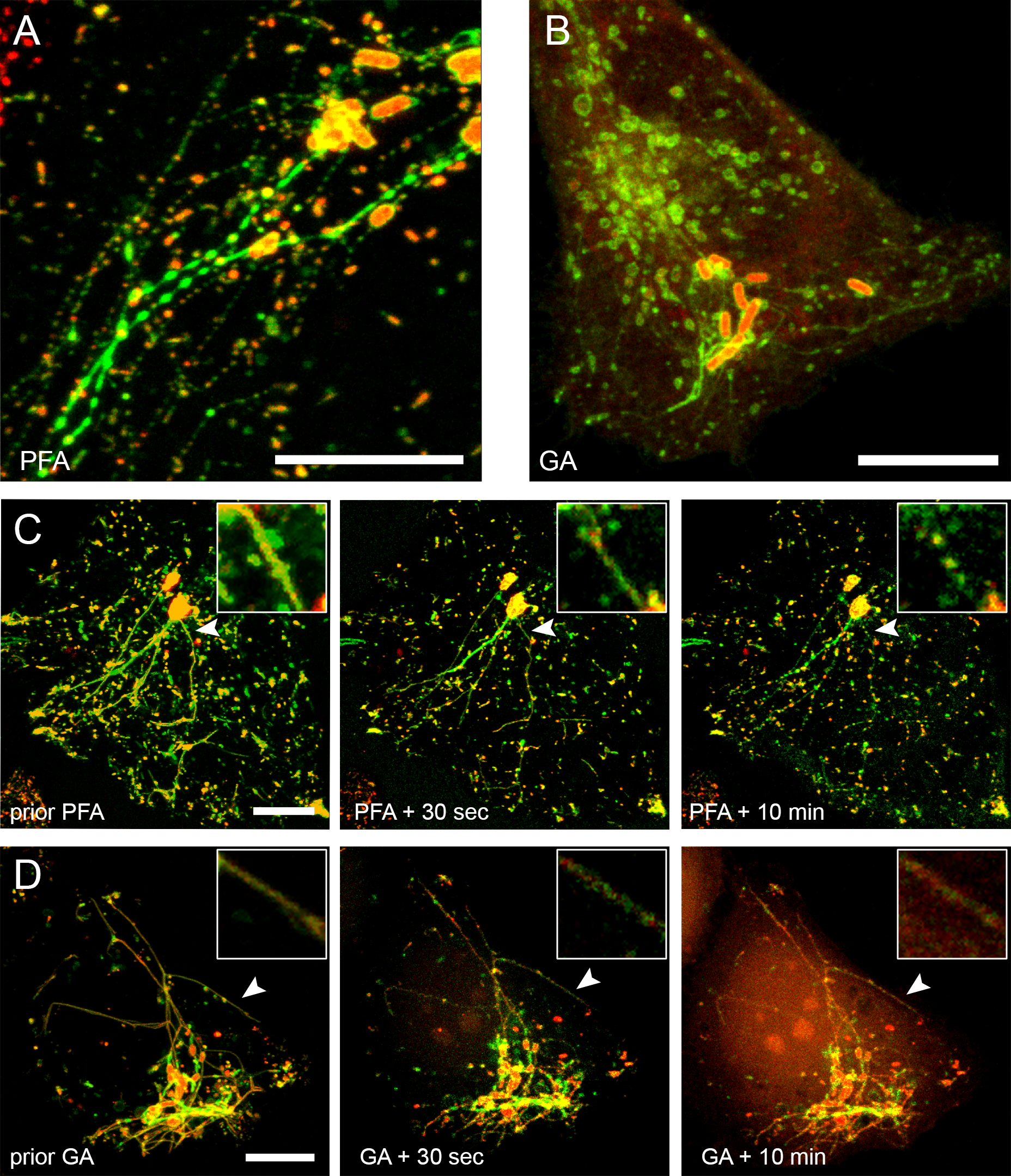

Supplement: S3 Fig — Effect of PFA and GA fixation on integrity of SIF. HeLa cells transfected with LAMP1-GFP (green) were infected with Salmonella sseF mutant constitutively expressing mCherry (red). At 2-5 h p.i. cells were pulse-chased with the fluid phase marker BSA-Rhodamine (red) for an additional labeling of SCV and SIF. Cells were washed at 8 h p.i. and fixed with 3% PFA (A) or 2.5% GA (B). Representative infected cells with pseudo-SIF phenotypes are shown. C), D) Live cell imaging of infected cells by CLSM was performed at 8 h p.i. Equal amounts of medium containing double concentration of PFA (C) or GA (D) were added to the cells directly on the microscope stage. The effect of fixation was monitored 30 sec and 10 min after addition. Note the vesiculation of membrane tubules after the addition of PFA, resulting in pseudo-SIF formation. GA fixation preserves the morphology of membrane tubules, but induces strong red autofluorescence. Scale bars, 10 µm. (TIF) [file pone.0115423.s003.tif]
